# Calixarene-Based Functional Fabric for Simultaneously Adsorptive Removal of Anionic and Cationic Dyes

**DOI:** 10.1021/acsomega.4c04109

**Published:** 2024-12-24

**Authors:** Egemen Ozcelik, Begum Tabakci, Mustafa Karaman, Mustafa Tabakci

**Affiliations:** † Department of Chemical Engineering, Konya Technical University, 42250 Konya, Türkiye; ‡ Department of Chemistry, Selçuk University, 42130 Konya, Türkiye

## Abstract

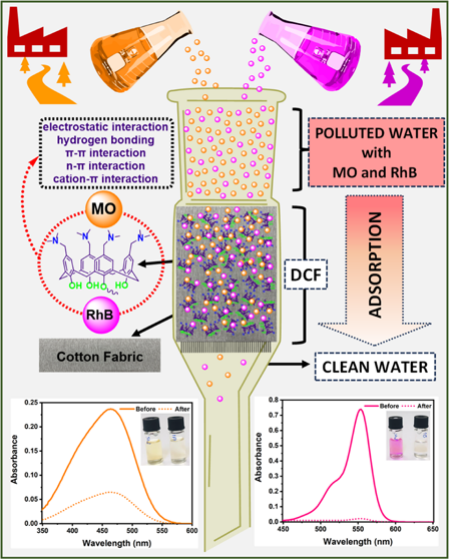

This study investigated the adsorptive properties of functionalized fabric containing dimethylaminomethyl calix[4]arene (DMAM-Calix) to remove anionic methyl orange (MO) and cationic Rhodamine B (RhB) dyes in aqueous media. Adsorption studies were performed using a filtration system packed with DMAM-Calix-functionalized fabric (**DCF**). The results revealed that the cationic and anionic structures work compatibly in a binary mixture medium. Hydrogen bonding, π–π, cation–π, *n*–π and electrostatic interactions between dye molecules and DMAM-Calix units of **DCF** were the main factors affecting the adsorption process. Experiments on real wastewater samples of unknown composition confirmed that the approach could successfully remove MO and RhB dyes from real water samples with high efficiency, especially for RhB. Isotherm and kinetic data for MO were mainly represented by the Langmuir model and pseudo-second-order kinetic model, respectively. The adsorption capacities of **DCF** were found to be about 4.7 mg g^–1^ for MO and 1.0 mg g^–1^ for RhB at pH 6.0, which were evaluated as satisfactory considering the first use of a calixarene-derived coated fabric as an adsorbent, the anionic–cationic dye selectivity of **DCF**, and the low cost and ease of application of the method.

## 1 Introduction

It is a fact that water, a resource that is vital to human survival, faces many difficulties, with water pollution emerging as one of the significant issues facing the globe today. Water pollution not only harms humans but also threatens the survival of other aquatic life. Pollution of water resources occurs in various ways, and industrial wastewater discharge is a typical example.^1^ Dyes, or coloring agents, are significant pollutants that have a wide range of applications in industries such as textiles, leather, paper, plastics, and printing.^2^ Recently, synthetic dyes have been produced and have gradually replaced natural dyes, especially in the fabric and textile sectors. Synthetic dyes generally consist of both ionic and nonionic dyes.^3^ Ionic dyes can be categorized as cationic dyes and anionic dyes (acid, reactive, and direct), while nonionic dyes can be categorized as vat and dispersed dyes.^4^ Furthermore, dyes classified based on their chemical structure are also widely present. These include azo, anthraquinone, indigo, nitroso, and triarylmethane dyes.^5,6^ While dyes make us bright colors, they also pose a severe hazard to life safety since they are discharged into waterways as industrial waste. The discharge of dye wastewater poses a significant threat to aquatic ecosystems and public health. Colored dyes in water can hinder the entry of sunlight, thus hindering the photosynthesis of underwater plants. The reduced levels of dissolved oxygen negatively affect the growth of aquatic life.^7^ The majority of dyes are complexes of organic compounds, including aromatics, amines, and trace heavy metals such as Cd, Cu, Pb, Zn, and Co. These components render dyes toxic.^8,9^ Thus, dyes can cause various health issues in humans, including methemoglobinemia, mental confusion, and potentially cancer.^10^ Given these harmful effects, it is imperative to address and treat the pollution of dyes in the water environment.^11^ To date, numerous strategies for removing organic dyes from industrial wastewater have been researched, including adsorption,^12^ advanced oxidation technology,^13^ photocatalytic techniques,^14^ and others. Because of its simplicity, economic feasibility, and excellent efficiency, adsorption is the most promising option among these.^15,16^

In the literature, many polymers and macromolecules can be used to remove ionic and neutral substances. Among them, calixarenes are a type of macromolecule that is synthesized through a condensation reaction between phenol and formaldehyde in alkaline conditions. They have a special place in host–guest chemistry due to their unique three-dimensional structure and their capability to interact specifically with different functional groups.^17^ Especially calix[4]arenes, which have a typical symmetrical bowl shape due to their lower-edge H-bond interactions, have attracted great interest as they can bind both organic and inorganic guests. Therefore, calixarenes remain popular building blocks in host–guest chemistry due to the aesthetic and practical prearranged nature of the host cavity for target compounds.^18^ The aromatic calixarene cavity exhibits CH-π, π–π, and cation-π interactions with the substrate, and it also serves as a convenient platform for adding the necessary substituents on the lower and upper edges.^19^ In fact, these properties have also been investigated as adsorbents. Many adsorption studies using calixarenes involve the immobilization of calixarenes on various support materials to remove a wide range of contaminant molecules.^19−20,40^ The performance of calixarene-bonded fabric in the adsorptive removal of Cr(VI) from aqueous media, which we reported in one of these studies, led us to investigate its usability in the removal of dyes, which are among the essential species causing water pollution.^41,42^ For this purpose, the functional adsorbent fabric (**DCF**) was prepared by first coating the surface of the bare fabric with poly(glycidyl methacrylate) (PGMA) by initiated chemical vapor deposition (iCVD) method and then immobilizing DMAM-Calix derivative over the epoxy ends on the surface. PGMA is a versatile coating material for many applications because of its pendant epoxy group, which can be attached to various chemical groups through a ring-opening reaction with nucleophiles.^43^ This nucleophilic ring-opening reaction makes PGMA a desirable material for various applications such as postpolymerization surface modifications, membrane science, and biochemistry.^44^ The purpose of coating PGMA on the bare fabric surface was to form epoxy units on the surface that could react with the hydroxyl groups of DMAM-Calix. In the literature, no study has shown that calixarene-attached fabric was used as an adsorbent for the adsorption of dyes. Therefore, to the best of our knowledge, this study is the first to investigate the structural effect of the functionality of calixarene on a fabric on dye adsorption. This study selected two dyes with different properties, namely methyl orange (MO) and Rhodamine B (RhB), as model dyes. The performance of the resulting calixarene-tethered fabric in the adsorptive removal of MO and RhB dyes was investigated.

## 2 Experimental Section

### 2.1 Reagents and Instrumentations

All the chemicals and reagents used in this investigation; *p-tert-*butylphenol (820250, Merck), formaldehyde (37%, 104002, Merck), sodium hydroxide (106462, Merck), diphenyl ether (820978, Merck), ethyl acetate (109623, Merck), acetic acid (glacial, 100066, Merck), aluminum chloride (801081, Merck), phenol (100201, Merck), toluene (108327, Merck), methanol (113351, Merck), dimethylamine (40%, 8.22033, Sigma-Aldrich), tetrahydrofuran (109731, Merck), sodium hydride (60%, 814552, Merck), tetrabutylammonium bromide (8.18839, Sigma-Aldrich), acetone (822251, Merck), dimethylformamide (103034, Merck), glycidyl methacrylate (779342, Sigma-Aldrich), di-*tert*-butyl peroxide (Luperox, 98%, 168521, Sigma-Aldrich), silicon wafer (100 p-type, 647764, Sigma-Aldrich), were acquired from Merck or Sigma-Aldrich as analytical or high-purity products and used directly without further purification. Analytical thin-layer chromatography (SiO_2_, Merck F254) was used to monitor the reactions. The present study confirmed all calix[4]arene structures using a Nicolet 380 FT-IR and a Varian 400 MHz NMR spectrometer, respectively. FT-IR spectrometer was worked with a range between 400 and 4000 cm^–1^ at a resolution of 4 cm^–1^ over 32 scans. The T80+ UV/vis spectrophotometer from PG Instruments was used for the adsorption studies.

### 2.2 Preparation of **DCF**

Initiated chemical vapor deposition of poly glycidyl methacrylate on fabric surfaces was carried out using glycidyl methacrylate (97%, 168521, Sigma Aldrich) as the monomer and ditertbutyl peroxide (Luperox, 98%, 168521) as the initiator. Details of the iCVD system and the deposition conditions are given elsewhere.^45^ During iCVD, cotton fabric was used as the substrate.

**Figure 1:**
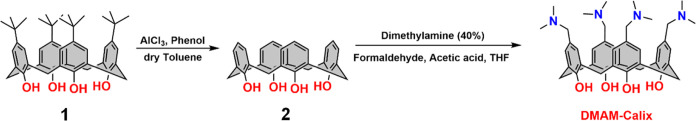
Synthesis scheme of DMAM-Calix.

**Figure 2:**
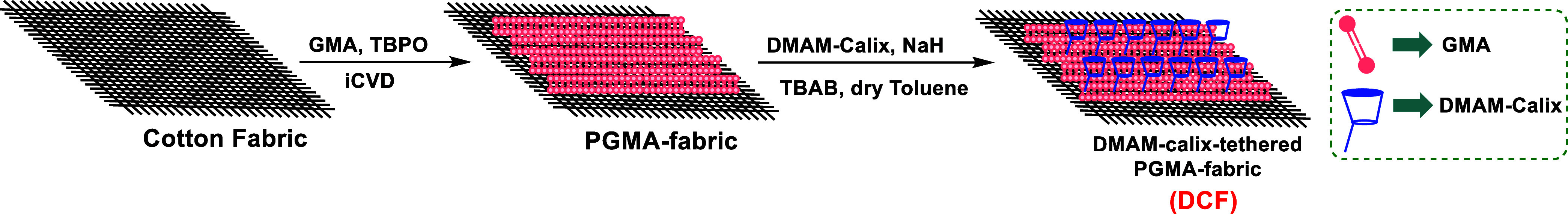
Preparation of DMAM-Calix-tethered PGMA fabric (**DCF**).

In our previous study, we synthesized a dimethylaminomethyl functionalized calix[4]arene derivative (DMAM-Calix) according to literature (Figure 1),^46^ and tethered it onto PGMA-modified cotton fabric by iCVD method (Figure 2).^41,42^ For this purpose, *p-tert-*butylcalix[4]arene (**1**) was synthesized from a phenol-formaldehyde condensation reaction under alkaline conditions. Its dealkylation with aluminum chloride and phenol in dry toluene gave calix[4]arene (**2**). The Mannich reaction of **2** was used to synthesize DMAM-calix. In this study, we have prepared a new one (**DCF**) using the same method from the literature to capture dye derivatives such as MO and RhB.

### 2.3 Adsorption Procedure

A 1 cm^2^ DMAM-Calix tethered PGMA fabric (**DCF**) was thoroughly compressed into a Pasteur pipet (length: 225 mm, outer diameter: 7 mm, capacity: 2.0 mL) to prepare the filtration system. Then, 5 mL of dye solutions containing RhB and MO were passed over the produced filter system (flow rate: ∼ 0.17 mL/min at 25 °C). The adsorption percentage was determined spectrophotometrically from the before and after concentrations of the solutions passing through the fabric. The adsorption percentage (Ads%) was determined using eq 1.
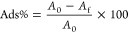
1where *A*_0_ and *A*_f_ are the initial and final absorbance values of the dye solution, respectively, the adsorbed amount of dye was determined by following eq 2.
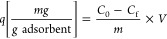
2Where *C*_0_ and *C*_f_ are the initial and final concentrations of dye solutions, respectively, *m* is the amount of adsorbent, and *V* is the volume of the solution. Desorption studies were also conducted to observe the reusability of the **DCF**. For this process, RhB was desorbed with methanol while MO was desorbed from **DCF** using NaCl/NaOH solution.

### 2.4 Fabrication Cost Analysis of **DCF**

An analysis of the cost of fabricating the adsorbent is a crucial study to assess the potential of the developed material for potential use in the treatment process once all other technical aspects have been met.^47^ The total production cost of **DCF** adsorbent was determined from the sum of the costs of preparation of DMAM-calix and PGMA fabric. The chemical cost and energy cost of the fabrication process were determined using the following equations.^47^
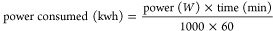
3
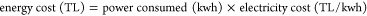
4
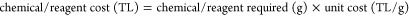
5

### 2.5 pH_PZC_ Determination

The point of zero charge (pH_PZC_) is a critical characterization parameter that is associated with the electrostatic interaction on the surface of adsorbents in relation to the pH of the medium.^48^ The pH_PZC_ was determined by varying the pH from 2 to 10 in a 25 mL solution of 1.0 × 10^–2^ (M) NaCl. The solution pHs were adjusted using NaOH and HCl solutions. Eighteen mg of **DCF** was interacted with NaCl solutions at different pHs for 1 h. Subsequently, the change in pH (ΔpH) was graphed against the initial pH (pH_o_). The pH_PZC_ refers to the point at which the initial pH (pH_o_) is equal to the final pH (pH_final_).^49^ According to the data presented in Figure S1, the pH_PZC_ (point of zero charge) of this material was determined to be 8.5. It suggests that the surface of **DCF** tends to become positively charged when the pH is lower than the pH_PZC_.

## 3 Results and Discussion

### 3.1 Synthesis and Characterization of **DCF**

**Figure 3:**
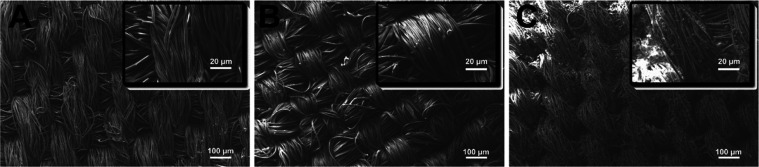
SEM images of (A) bare fabric, (B) PGMA-coated fabric, and (C) **DCF**.

In our previous study,^41^ a DMAM-Calix functionalized fabric was used as an adsorbent for Cr(VI) ion removal, and the result was successful. The functional fabric (**DCF**) was reproduced in this study, and its adsorptive properties were investigated for removing anionic (MO) and cationic (RhB) dyes in aqueous media. For this purpose, **DCF** was obtained using the method given in the literature.^41^Figure 3a shows the SEM images of untreated cotton fabric fibers, and Figure 3b shows the SEM images of fabric fibers formed by coating the surface with PGMA groups by the iCVD method. The changes in Figure 3b compared to Figure 3a indicate that PGMA forms a smooth coating on the surface. Figure 3c shows the SEM image of the fabric fibers resulting from the ring opening reaction of the glycidyl groups of the PGMA with the hydroxyl groups of the DMAM-Calix structure. According to this, it is understood from the significant changes in the cotton fabric surface that this formation occurs homogeneously. However, a rough surface is formed in some regions because DMAM-Calix is a relatively large molecule.

### 3.2 Adsorption Studies

#### 3.2.1 Effect of Solution pH

The pH of the dye solution has a substantial influence on the adsorption process, as it impacts both the extent of ionization of the adsorptive molecules and the surface characteristics of the adsorbent.^48,50^ The chemical bonding of the dyes in the solution and the ionization state of the functional groups of the sorbent are both influenced by the pH, which in turn affects the adsorption of ionic dyes on adsorbents.^51^ The influence of pH on the adsorption of dye compounds was investigated in the following manner: Solutions containing 2.5 mg/L of MO and 1.0 mg/L of RhB dye were prepared at different pH levels (2, 3, 4, 5, 6, 7, 8, 9, and 10). These solutions were then subjected to treatment with 18 mg of **DCF** at a temperature of 25 °C for 1 h. The highest level of MO dye removal using **DCF** was observed at a pH of 2.0, with an uptake efficiency of 96.34 (±1.78) %. The plot (Figure S2) demonstrates a significant decrease in removal efficiency from pH 2.0 to 10.0. To ensure the appropriate disposal of treated effluent, we have concluded that a solution pH of 6.0, with a removal efficiency of 90.24 (±1.33)%, is the optimal choice. It is because there is no significant difference in removal efficiency between 96.34 (±1.78) % and 90.24 (±1.33) %.^50^ From the results given in Figure S2, it was found that, in general, acidic and even neutral conditions are more favorable for the adsorption of MO. This result can be attributed to the abundance of H^+^ ions in the regions of the MO molecule, leading to the formation of the quinoid form of MO at low pH values. It generates an electrostatic attraction toward the nitrogen and oxygen parts on the surface of the **DCF**, which function as electron donors.^36^ As the pH of the solution increased, the concentration of positive ions decreased, which could result in a decrease in adsorption due to the weakened attraction between the ions and the surface. In other words, this decrease can be explained by the fact that MO, when exposed to high pH levels, becomes negatively charged and potentially competes with OH^–^ ions for binding sites that are available. However, hydrogen bonding, cation-π, *n*-π, and π–π dispersive interactions (resulting from the presence of aromatic rings in MO) play an essential role in the binding of MO to the surface of **DCF**.^52^ Additionally, the significant decrease at pH 9.0 may be attributed to the further intensification of electrostatic repulsion, as the **DCF** surface becomes negative at pH levels greater than pH_pzc_ (8.5). Regarding RhB, there was minimal adsorption observed under pH < 4.0 conditions, which can be attributed to the complete cationic state of RhB. At pH > 4.0, a significant increase in adsorption was observed due to the zwitterionic form of RhB (positive and negative charges are located at = N^+^ and COO^–^, and the negative side at COO^–^ can interact with positively charged **DCF** surfaces) and a decrease in H^+^ ions. However, at pH > 7.0, a decrease in adsorption was observed, probably due to the growth of RhB by forming dimers and aggregates.^38^ Furthermore, for comparison purposes, DMAM-calix unmodified cotton fabric fibers were also used as adsorbents for MO and RhB. As shown in Figure S2, the bare fabric did not exhibit significant adsorption at any pH. This result clearly demonstrated the importance of the structural feature of calixarene content in the **DCF** structure for high dye removal.

#### 3.2.2 Effect of Initial Dye Concentration

The initial concentration of the analyte can be correlated with the adsorption capacity of an adsorbent. Typically, when the active site on the surface of the adsorbent becomes saturated, the percentage of removal decreases as the initial concentration increases. On the other hand, if the active site is not fully occupied, the percentage of dye removal increases as the initial dye concentration increases. It is because the high concentration of dye creates a significant force that drives the mass transfer toward adsorption.^53^

**Figure 4:**
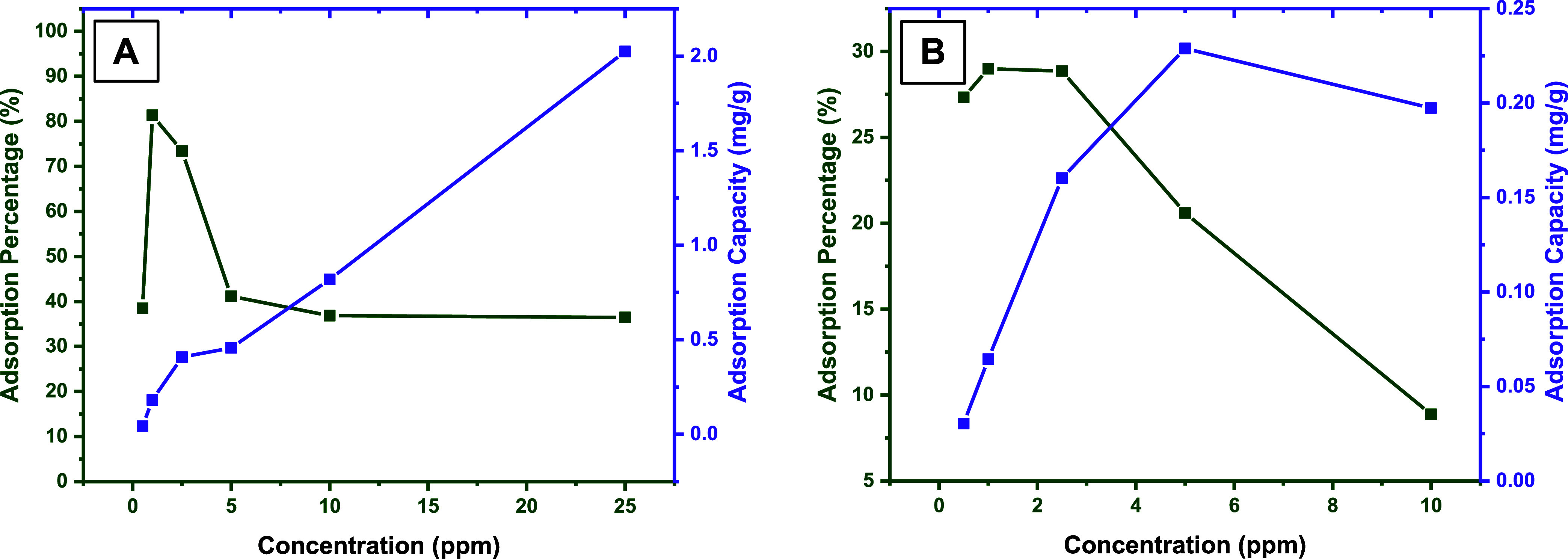
Effect of the initial concentration on the adsorption of (A) MO and (B) RhB by **DCF**.

The effect of dye concentration on adsorption percentage and capacity of **DCF** was determined using MO (in the range of 0.5–25 mg/L) and RhB (in the range of 0.5–10 mg/L) solutions. According to the results given in Figure 4a, in the case of MO, it was observed that the adsorption percentage decreased as the concentration increased after 1 mg/L, whereas the adsorption capacity continued to increase. When the MO concentration reached 25 mg/L, the adsorption capacity was calculated to be about 2 mg/g. When the MO structure^38^ is examined, it is seen that it has parts capable of hydrogen bonding, electrostatic, and π-π interactions. Therefore, thanks to these parts, DMAM-Calix units carried by the adsorbent can form both endo and exo complexes with MO molecules.

On the other hand, similar trends were observed in the case of RhB. From the graphs given in Figure 4b, it can be seen that the adsorption percentage increases up to 1 mg/L, as in the case of MO, and then decreases. Moreover, there is a continuous increase in adsorption capacity with increasing concentration. In the case of RhB,^38^ it can be concluded that it has moieties capable of hydrogen bonding, π-π, cation-π, and electrostatic interactions, and all of them, except electrostatic interactions with DMAM-Calix units, make a joint contribution to RhB adsorption. Here, the electrostatic interactions may have negatively affected RhB adsorption because they caused repulsion between the cationic RhB and the quaternary ammonium cation moieties of DMAM-Calix units, which were slightly formed under the experimental pH conditions.

#### 3.2.3 Binary Mixture Studies

**Figure 5:**
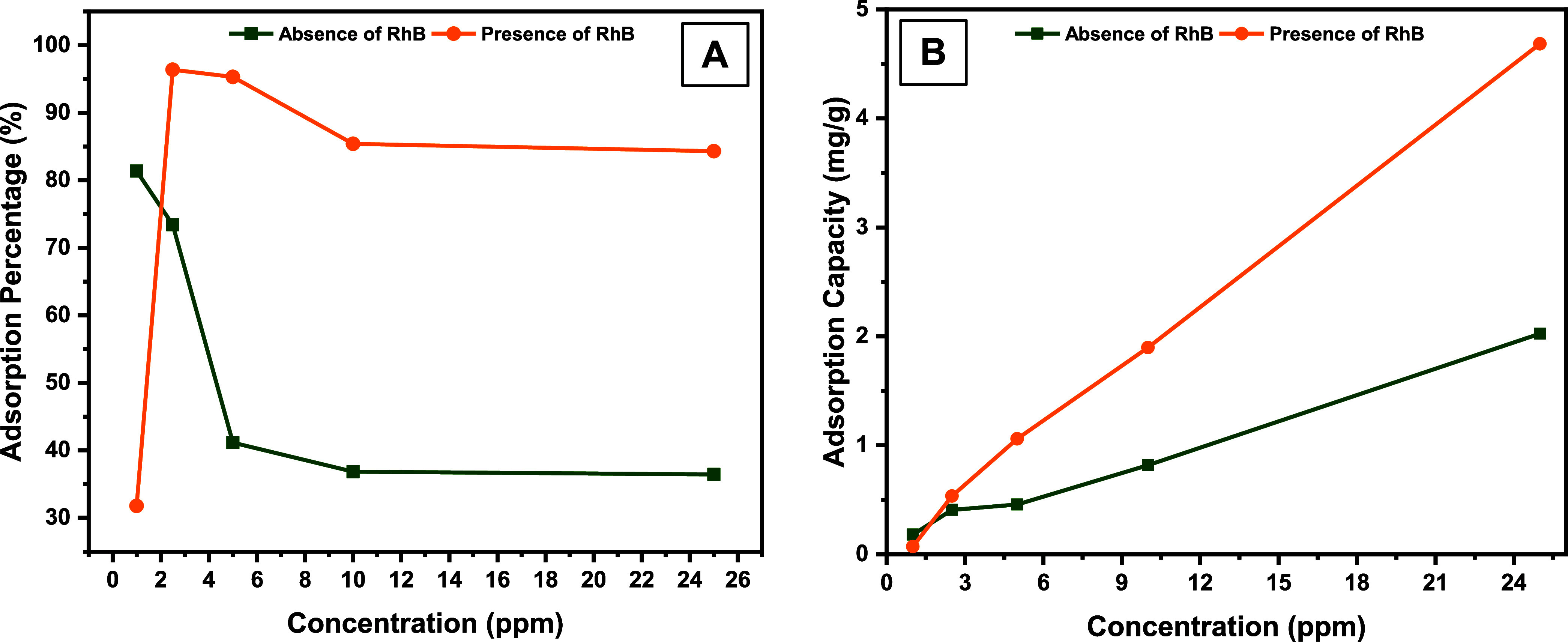
Effect of RhB (*C*_o_ = 1.0 mg/L) on the adsorption of MO (*C*_o_ = 1.0 - 25 mg/L) by **DCF** in terms of (A) adsorption percentage and (B) adsorption capacity.

Their simultaneous adsorption on **DCF** was investigated using combined MO and RhB dye solutions. The simultaneous adsorption of MO and RhB was studied in two binary systems to see both the effect of RhB presence on MO removal and the effect of MO presence on RhB removal. In one of them, the initial concentration of RhB was kept constant at 1 mg/L, while the initial concentration of MO was varied from 1 to 25 mg/L. In the other, the initial concentration of MO was kept constant at 2.5 mg/L, while the initial concentrations of RhB varied from 0.5 to 10 mg/L. Figure 5a,5b show the adsorption percentage and adsorption capacity values obtained from this study for MO and RhB, respectively. When the graphs presented comparatively for the first binary system in Figure 5 are examined, it is observed that the adsorption percentage and capacity value for 1 mg/L MO in the presence of RhB is lower than the value obtained for 1 mg/L MO in the absence of RhB. However, at concentrations above 1 mg/L MO, it was observed that the adsorption capacity and percentage in the presence of RhB were higher than in the absence of RhB. So, when the concentration of 25 mg/L MO was reached, the adsorption capacity increased from 2 to 4.7 mg/g.

**Figure 6:**
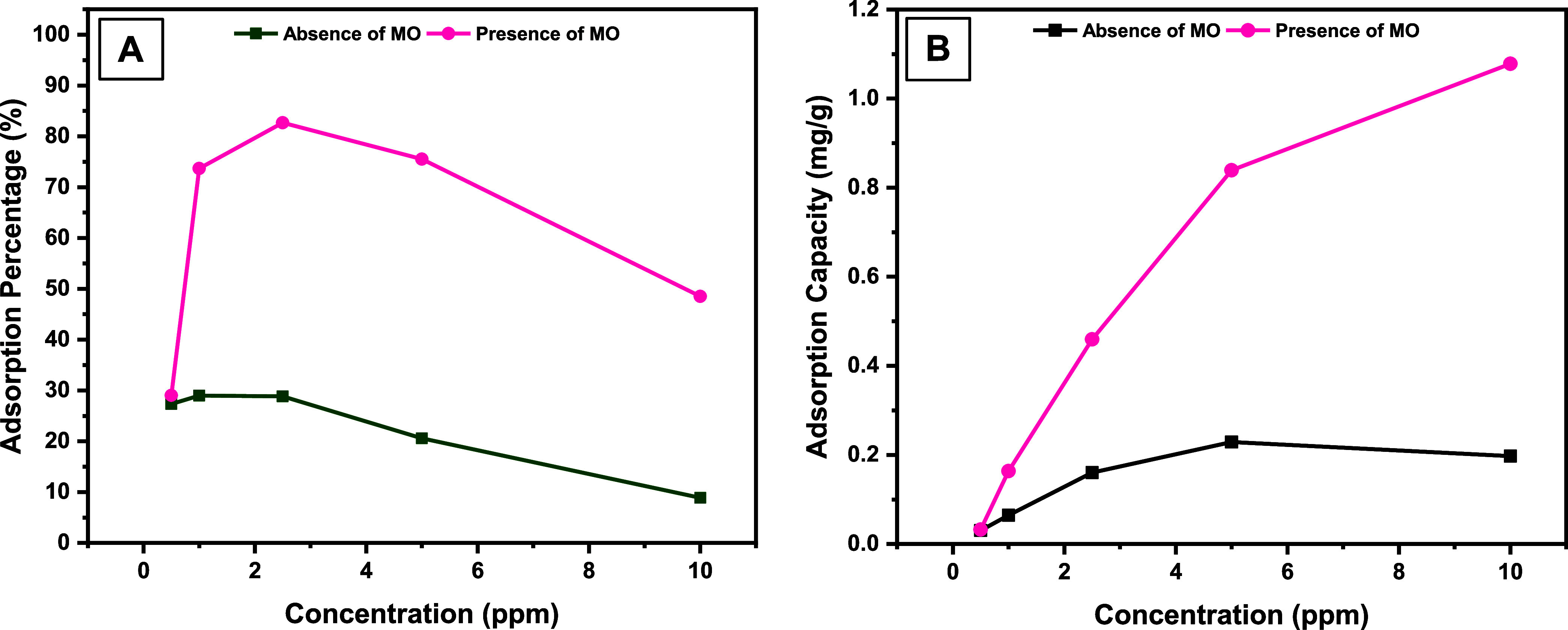
Effect of MO (*C*_o_ = 2.5 mg/L) on the adsorption of RhB (*C*_o_ = 0.5–10 mg/L) by **DCF** in terms of (A) adsorption percentage and (B) adsorption capacity.

The graphs from the other binary system presented in Figure 6 indicate almost the same trend. So, it was found that the percentage (Figure 6a) and capacity (Figure 6b) of RhB adsorption in the presence of MO increased at all concentration values compared to the values in the absence of RhB. So, when the concentration of RhB was 10 mg/L, the adsorption capacity increased from 0.2 to 1 mg/g.

**Figure 7:**
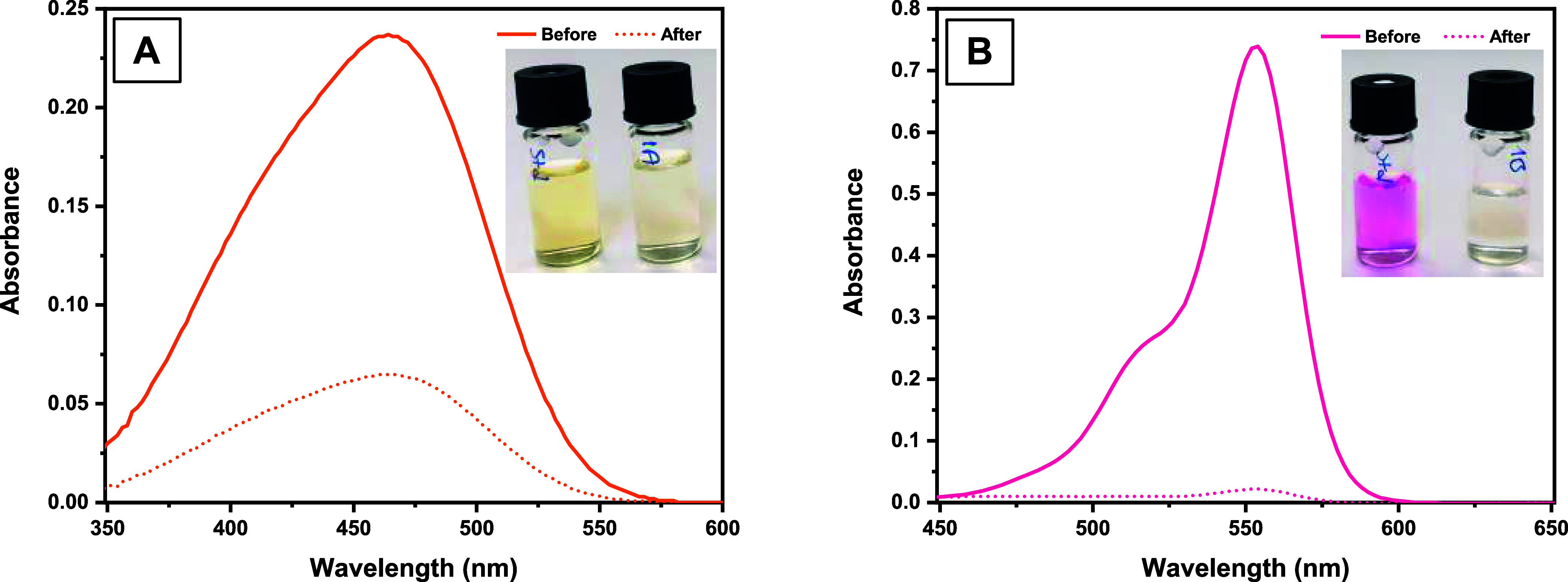
UV spectra of (A) MO and (B) RhB solutions after and before adsorption by **DCF** ([*C*_o_] = 2.5 mg/L).

**Figure 8:**
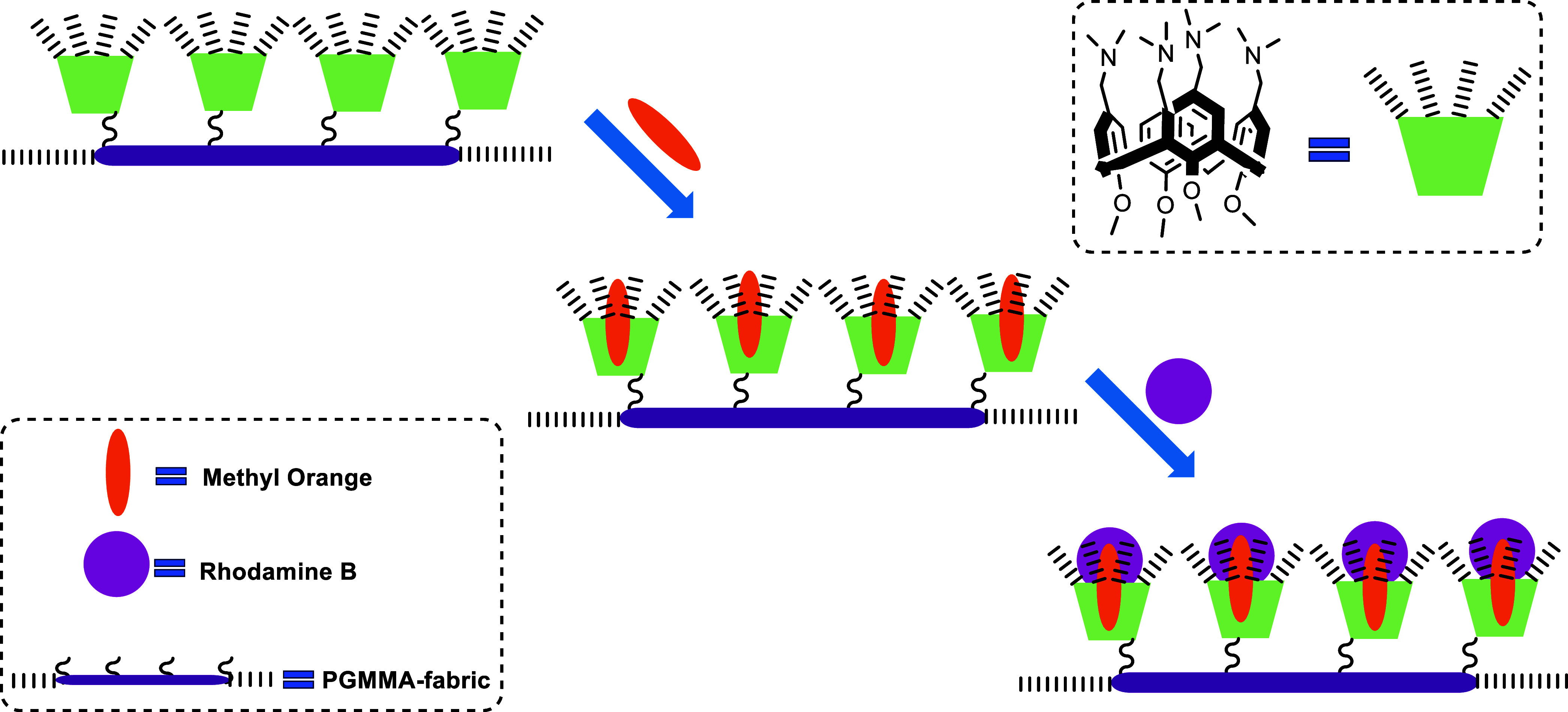
A possible adsorption mechanism of MO and RhB by **DCF**.

The increase in adsorption amounts in both cases indicates that the cationic and anionic structures work in harmony with each other in a binary mixture environment. To further clarify this, the dye solutions were passed over **DCF** instead of being applied together, respectively, and their UV spectra were compared (Figure 7). First, 2.5 mg/L MO solution was passed through the **DCF**, followed by 2.5 mg/L RhB solution. The UV spectra observed as a result of desorption revealed that approximately 70% removal occurred after the MO solution was passed (Figure 7a). In addition, almost all of the RhB was captured by **DCF** (Figure 7b). These findings suggest that the complexation of DMAM-Calix units on **DCF** with MO leads to a more efficient capture of RhB. Therefore, RhB may have been completely captured at this concentration level thanks to the complex development of new interaction sites with the MO-DMAM-Calix complex. As a result, it was seen that MO and RhB adsorption both increased in the binary system, which suggested a synergistic adsorption occurring.^54^ A possible adsorption mechanism is given in Figure 8. Herein, noncovalent interactions such as hydrogen bonding, *n*-π, cation-π, π–π, and electrostatic interactions mainly play an essential role in the adsorption mechanism of both MO and RhB onto **DCF**.

#### 3.2.4 Effect of Coexisting Ions

**Figure 9:**
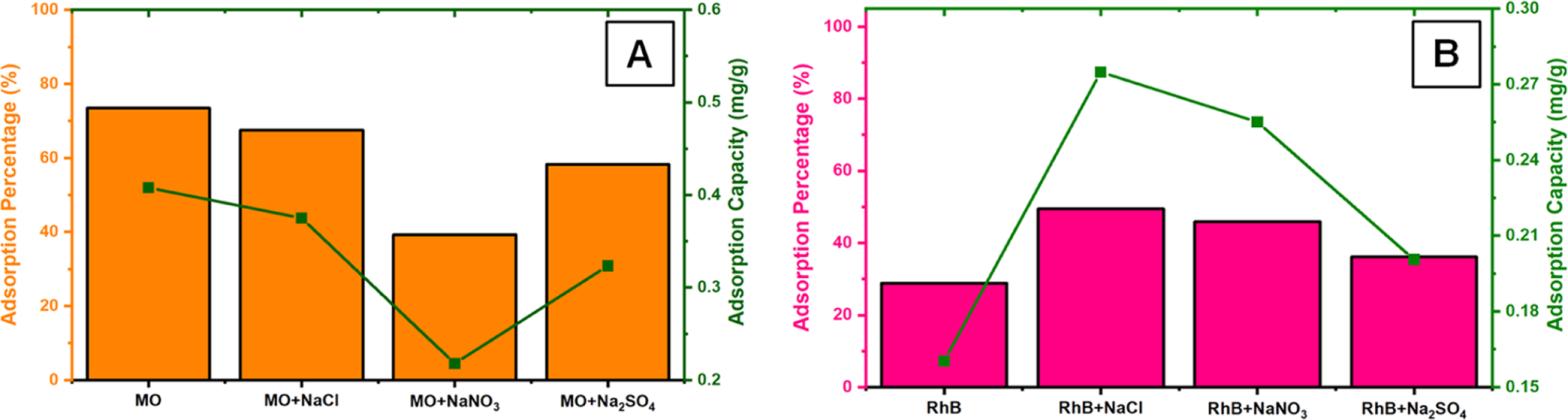
Effect of coexisting ion (*C* = 25.0 mg/L) on the (A) MO and (B) RhB 2.5 mg/L MO concentration by **DCF** (*C*_o_ = 2.5 mg/L).

Environmental waters contain competitive anions, including chloride, nitrate, and sulfate. To see the effect of these anions on the adsorptive removal of MO and RhB by **DCF**, a mixture of dye solution (2.5 mg/L) and anion solution (25 mg/L) was applied to packed **DCF**. Figure 9a,9b show the adsorption amounts obtained for RhB and MO, respectively. Accordingly, the adsorption amount for MO was negatively affected by the presence of various anions. Considering the anionic nature of MO, this may be due to the competitive coordination of anions on **DCF**, which participate in the electrostatic interaction in the adsorption process.^55^ The results obtained in the case of RhB also support this assessment. Cationic RhB adsorption was positively affected by the presence of various anions, and the amount of RhB adsorption increased in contrast to that of anionic MO. On the other hand, this increase in RhB adsorption is consistent with the phenomenon of anionic MO.

#### 3.2.5 Effect of Ionic Strength

**Figure 10:**
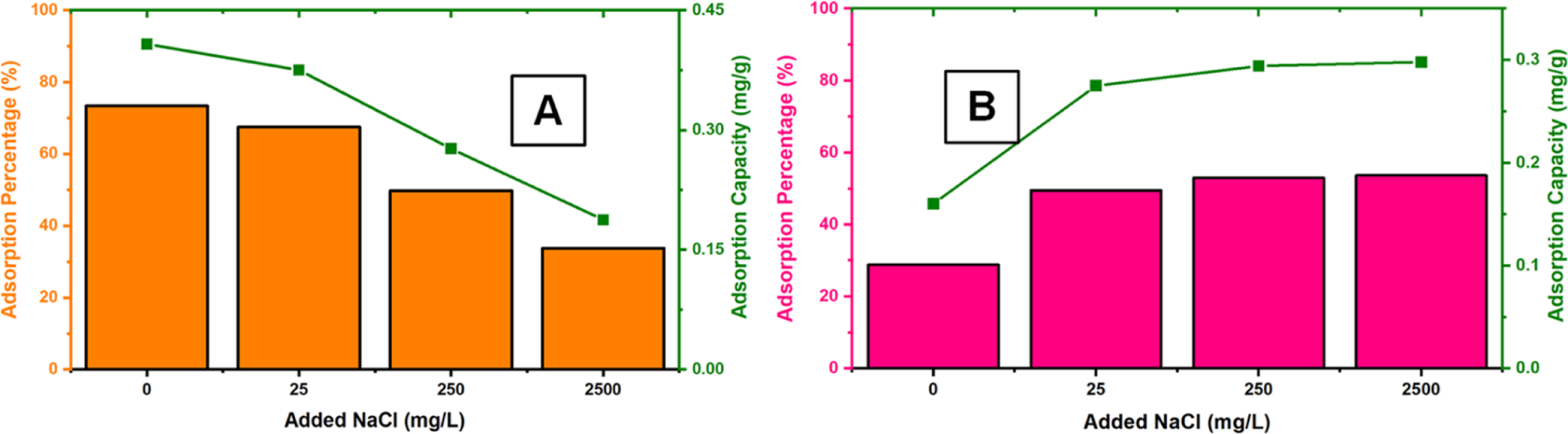
Effect of ionic strength on the adsorption of MO by **DCF** (*C*_o_ = 2.5 mg/L).

A mixture of dye solution (2.5 mg/L) and various NaCl solutions (25–250–2500 mg/L) was applied to the packed **DCF**, and the effect of ionic strength on MO and RhB adsorption was studied. According to the adsorption data in Figure 10a,b for RhB and MO, the adsorption of MO decreases steadily with the increase in NaCl concentration, just like the effects of other anions. It seems to result from **DCF**’s preference for anionic species, as in previous experiments. In the case of RhB, the adsorption behavior of **DCF** was again reversed. In the presence of NaCl in different concentrations, RhB adsorption was positively affected; the adsorption amount increased up to 25 mg/L and remained stable afterward. According to the results obtained, it can be said that ionic forces have an essential effect on the removal of RhB and electrostatic interactions, as π-π interactions play an essential role in the adsorption mechanism.

#### 3.2.6 Recovery Studies

**Figure 11:**
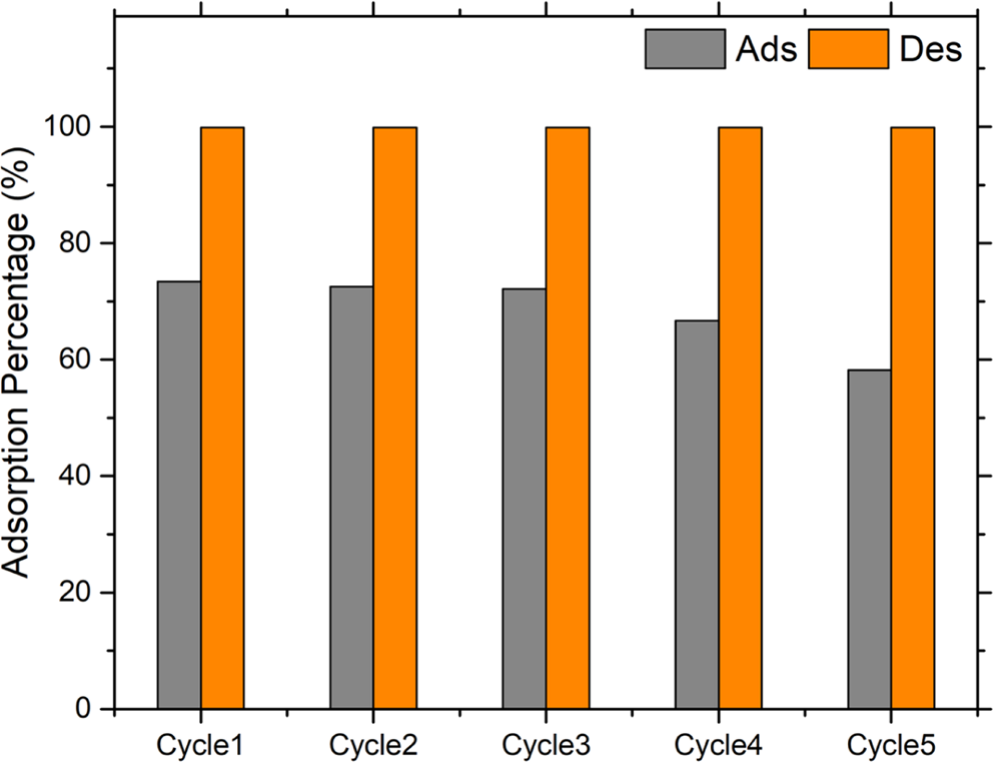
Recyclability of **DCF** for the MO adsorption (*C*_o_ = 2.5 mg/L).

Investigation of the adsorption ability of **DCF** for these two dyes showed that higher adsorption capacity was achieved for MO than for RhB. This result indicates that **DCF** is selective toward the anionic structure. Accordingly, adsorption–desorption cycling studies for anionic MO dye were carried out to evaluate the reusability of **DCF**. This experiment was repeated five times using 18 mg of adsorbent dispersed in 5 mL of 2.5 mg/L MO solution (pH ∼ 6). Then, the used **DCF** adsorbent was washed several times with 1.0 × 10^–2^ (M) NaOH and oven-dried. Figure 11 depicts a column diagram of five cycles of MO adsorption by **DCF**, demonstrating that the reuse rate steadily declines after five reuse experiments due to **DCF** loss. However, after five recycling studies, the reuse rate remained above 60%, demonstrating that **DCF** is acceptable for the cyclic adsorption of MO.

#### 3.2.7 Adsorption Isotherms

Langmuir, Freundlich, and Temkin isotherms^41^ were used to study the nature of MO adsorption with **DCF** in aqueous media. The obtained isotherm plots are given in Figure S3. Table 1 summarizes the adsorption constants obtained from the adsorption isotherms. The *R*^*2*^ value and *q*_0_ value for MO were determined as 0.877 and 0.887 mg/g, respectively, with Langmuir isotherm. In comparison, the *R*^*2*^ value and *K*_F_ value for MO were determined as 0.899 and 0.377 mg/g, respectively, with Freundlich isotherm. Although slightly higher for the Freundlich isotherm, there is no significant difference between the *R*^*2*^ values, indicating that MO adsorption is suitable for both Langmuir and Freundlich. Moreover, the 1/*n* value of the Freundlich isotherm (0.376) is close to zero, indicating a more heterogeneous multilayer surface. The Temkin isotherm expresses the heat of adsorption of the adsorbent molecule on the surface. The calculated *B*_T_ value of 3.82 kcal/mol indicates that the mechanism adopts physisorption since the heat of adsorption is less than 10 kcal/mol.

**Table 1:** Adsorption Isotherm Parameters for Adsorption of MO onto **DCF**

**isotherm models**	Langmuir		Freundlich		Temkin	
equations	*y =* 1.127*x* + 1.355		*y =* 0.376*x* – 0.424		*y* = 0.155*x* + 0.434	
isotherm parameters	*R*^*2*^	0.877	*R*^*2*^	0.899	*R*^*2*^	0.847
	*q*_0_ (mg/g)	0.887	*K*_F_ (mg/g)	0.377	*B*_T_ (kcal/mol)	3.824
	*b* (L/mmol)	272	*n*	2.66		
	*R_L_*	0.991	1/*n*	0.376	*A* (L/mg)	16.46

As a result of the adsorption experiments, the adsorption capacities of **DCF** were determined to be approximately 4.7 mg/g and 1.0 mg/g for MO and RhB, respectively. While it is not feasible to make a direct comparison between **DCF** and other sorbent materials due to variations in experimental conditions and structural disparities, Table 2 provides a comparison with adsorbents that contain calixarene (including resorcinarene). According to the data in Table 2, although the adsorption capacity values of **DCF** seem to be relatively low, considering the use of a calixarene derivative coated fabric for the first time as an adsorbent for dyes, the anionic–cationic dye selectivity of **DCF**, the cheap and easy applicability of the method, the obtained capacity values can be considered satisfactory.

**Table 2:** Comparison of Adsorption Capacities of **DCF** for MO and RhB with Other Sorbents

**no**	**calixarene derivative**	**structure/supportmaterial**	**experimental conditions**	**used dyes**	**adsorption capacity**	**refs**
1	calix[4]arene joined by a linear benzidine linker	covalent organic frameworks	*C*_0_= ∼16 mg/L (MO), *C*_0_= ∼24 mg/L (RhB), adsorbent mass = 7.5 mg, 25 °C	MO and RhB (and others)	N/A (MO) and 25 mg g^–1^ and 40 mg/g RhB	(25)
2	pyrazine-2-carboxylate functionalized calix[8]arene		*C*_0_= ∼6.5 mg/L (MO), adsorbent mass = 25 mg, 25 °C	MO (and others)	N/A	(27)
3	tetrakis-(*N*,*N*-diethy-l, 2-aminoethylamidomethoxy)-calix[4]arene	Amberlite XAD-4 resin	*C*_0_= ∼6.5 mg/L (MO), adsorbent mass = 50 mg, 25 °C	MO (and others)	350 mg/g MO	(34)
4	amidoamine calix[4]resorcinarene	polymer	*C*_0_= ∼32 mg/L (MO) adsorbent mass = 1.0 mg, N/A	MO (and others)	373 mg/g MO	(19)
5	morpholinomethylcalix[4]arene	silica	*C*_0_= 5 mg/L (MO) adsorbent mass = 40 mg, 25 °C	MO (and others)	175.4 mg/g MO	(36)
6	sulfonate modified tetraethynyl pyrencalix[4]resorcinarene	porous organic polymer	*C*_0_= 50 mg/L (RhB and MO) adsorbent mass = 25 mg, 25 °C	MO and RhB (and others)	N/A (MO) and 183, 2653, 1132, and 1796 mg/g (RhB)	(37)
7	floronaphtalene calix[4]resorcinarene	porous organic polymer	*C*_0_= 50 mg/L (RhB and MO) adsorbent mass = 5 mg, 25 °C	MO and RhB (and others)	862 mg/g (MO) and 2433 mg/g (RhB)	(38)
8	dimethylaminomethyl functionalized calix[4]arene	cotton fabric	*C*_0_= 2.5 mg/L (MO) and 1.0 mg/L (Rhb) adsorbent mass = 18 mg, 25 °C	MO and RhB	4.7 mg/g MO and 1.0 mg/g RhB	this study

#### 3.2.8 Effect of Temperature

MO adsorption onto **DCF** was tested at several temperatures, including 298, 308, 318, and 328 K. The adsorption yields were determined by applying eq 1, and the corresponding data is presented in Figure S4a. As depicted in Figure S4a, the adsorption yield exhibits a negative correlation with rising temperature. It is widely recognized that the efficiency of adsorption is typically inversely related to the temperature of the adsorption medium.^56^ This phenomenon means that low temperatures are also valid for MO adsorption onto **DCF**.

The equations used for the calculation of thermodynamic parameters, including standard adsorption free energy (Δ*G*^o^), enthalpy (Δ*H*^o^), and entropy (Δ*S*^o^), are represented by eqs 6 and 7:^17^
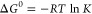
6
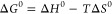
7Herein, *R* represents the ideal gas constant, which has a value of 8.314 kJ kmol^–1^ K^–1^. *K* represents the equilibrium constant, which is the ratio of the concentration of adsorbed MO to the concentration of MO remaining in the solution. *T* represents the temperature measured in Kelvin.

According to the Van’s Hoff equation (eq 8)
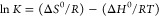
8where Δ*S*^*o*^ and Δ*H*^*o*^ are changes in entropy and enthalpy of adsorption, respectively.

Figure S4b displays a plot of ln *K* versus 1/*T*. The values of Δ*S*^o^ and Δ*H*^o^ were determined by analyzing the slope and intercept of the Van’t Hoff plots, as shown in Table 3. The negative Δ*H*^o^ value indicates that adsorption occurred with the release of heat, making it an exothermic process. The Δ*G*^o^ values exhibited a negative trend and demonstrated a positive correlation with increasing temperature. Consequently, the adsorption of MO onto **DCF** was found to be spontaneous and exhibited an upward trend with the rise in adsorption temperature. The negative values of Δ*S*^o^ indicate a reduction in randomness at the solid-solution interface during the adsorption of MO onto **DCF**.^57^

**Table 3:** Thermodynamic Parameters for Adsorption of MO onto **DCF**

Δ*H*^o^ (kJ mol^–1^)	Δ*S*^o^ (kJ mol^–1^ K^–1^)	*T* (K)	Δ*G*^o^ (kJ mol^–1^)
–27.97	–0.075	298	–2.51
		308	–1.99
		318	–1.09
		328	–0.29

#### 3.2.9 Adsorption Kinetics

Investigating the effect of contact time on the adsorption capacity of **DCF** is essential in designing an adsorption process, as it helps establish an adsorption equilibrium.^58^ Pseudo-first-order (eq 9), pseudo-second-order (eq 10), and intraparticle diffusion (eq 11) linear modeling equations given below are used to determine the adsorption rate.^59^
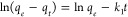
9
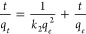
10
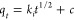
11

In the above equations, the equilibrium adsorption capacity (mg/g) is denoted as *q*_*e*_, *q*_*t*_ designates adsorption capacity (mg/g) at time *t*, and *k*_1_ (g/(mg·min)), *k*_2_ (g/(mg·min)) and, *k*_id_ (mg/g·min^1/2^) signify the rate constants of pseudo-first-order, pseudo-second-order, and intraparticle diffusion model, respectively. Pseudo-first-order and pseudo-second-order kinetics designate the physical and chemisorption, respectively. In eq 11, the parameter “*c*” represents the intercept obtained from the linear regression of the intraparticle diffusion model. This intercept indicates the depth of the boundary layer. A more considerable value of “*c*” signifies a more significant impact of the boundary layer on the adsorption process. In the intraparticle diffusion model, the rate of the process is solely determined by pore diffusion when the linear regression of the experimental data intersects the origin of the plot. On the other hand, in cases where linear data fitting reveals two linear lines, the initial line corresponds to diffusion taking place in the boundary layer. In contrast, the subsequent line corresponds to diffusion occurring within the particles. In the second scenario, the process of intraparticle diffusion is not the sole factor that determines the rate.

The contact time effect on the adsorption of MO onto **DCF**, all kinetic models were shown in Figure S5, and the relevant kinetic parameters were collected in Table 4. The pseudo-first-order and pseudo-second-order kinetic parameters revealed that the second-order kinetic model was more feasible in terms of correlation coefficients of the first and second-order kinetic model, and the adsorption capacity was closer to the experimental equilibrium adsorption capacity than the first-order kinetic model for the adsorption of MO on **DCF**. In the intraparticle diffusion kinetic model, the linearly fitted models did not intersect the origin of the plot, and the value of “*c*” is also nonzero. It indicates that the rate-determining step in this adsorption procedure is not governed by intraparticle diffusion.^47^

**Table 4:** Kinetic Parameters for Adsorption of MO onto **DCF** (*C*_o_ = 2.5 mg/L, *q*_exp_ = 4.664)

**kinetic models**	pseudo-first-order		pseudo-second-order		intraparticle diffusion	
kinetic parameters	*R*^*2*^	0.9761	*R*^*2*^	0.9988	*R*^*2*^	0.9713
	*q*_*e*_ (mg/g)	2.213	*q*_*e*_ (mg/g)	4.892	*C* (mg/g)	2.9806
	*k_1_* (g/(mg·min))	0.0057	*k*_2_ (g/(mg·min))	0.0054	*k*_id_ (mg/g·min^1/2^)	0.0685

#### 3.2.10 Spiked Real Wastewater Application

**Figure 12:**
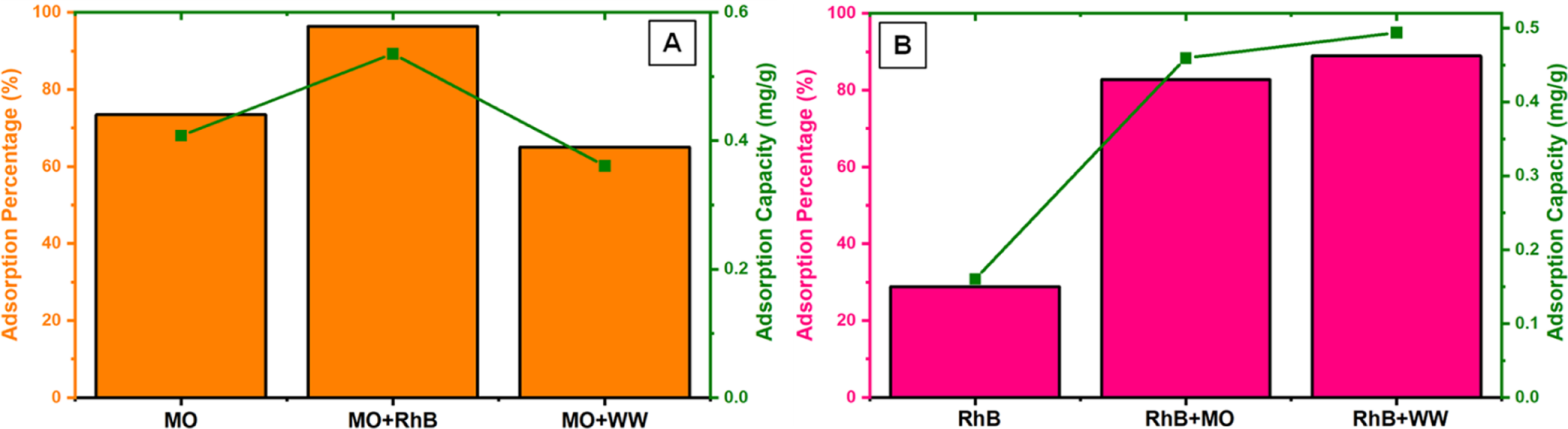
Graphs regarding adsorption of (A) MO and (B) RhB by **DCF** compared with spiked wastewater samples (*C*_0_ = 2.5 mg/L).

To test the adsorptive efficiency of **DCF** for MO and RhB in real wastewater samples of unknown composition, spiked water samples were generated by adding dye solution (2.5 mg/L) to the real wastewater sample. After the samples were applied to the packed **DCF**, the results were observed for MO and RhB in Figure 12a,12b, respectively. When the data on MO removal were analyzed, it was observed that 65% removal was achieved, although some decrease in adsorption was observed compared to the single and binary mixture experiments. In the case of RhB, an increase in adsorption was realized in contrast to MO. Compared to previous experiments, these results showed that the removal of RhB was high in the presence of MO and the unknown content wastewater sample. The results show that the method can be successfully applied to remove MO and RhB dyes in real water samples with high efficiency, especially for RhB.

### 3.3 Cost Analysis

A cost analysis was conducted to examine the economic aspect of field application.^47^ The cost of producing the DCF adsorbent must be cost-effective to enable its practical implementation on a large scale. The cost of preparing DCF adsorbent encompasses both the chemicals and energy expenses. The specific cost breakdown of DCF adsorbent in terms of cost per gram of adsorbent is presented in Table S1 of the Supporting Information, utilizing eqs 3, 4, and 5. The calculations revealed that the total cost of preparing the DCF adsorbent is approximately $2.0/g.

## 4 Conclusions

In this study, we took a different approach than previous studies in the literature that focused on removing dye compounds. Instead of following conventional methods, a filtration system was created by immobilizing a calix[4]arene derivative on a fabric. At this point, the present study was the first example of such a system in the literature. In this system, adsorption experiments were carried out for anionic MO and cationic RhB, and it was observed that DCF has significant adsorption properties to both anionic and cationic dyes. Hydrogen bonding, π-π, cation-π, *n*-π, and electrostatic interactions played important roles in adsorption in all complexation mechanisms. While **DCF** exhibited the highest adsorption for MO in the single solution study, the adsorption of both MO and RhB was enhanced due to the synergistic effect in the binary solution studies. Recovery studies revealed that **DCF** maintained its efficiency for at least five cycles. In the real wastewater samples, MO adsorption properties slightly decreased, but RhB adsorption properties increased significantly. The adsorption capacities of **DCF** were found to be about 4.7 mg/g for MO and 1.0 mg/g for RhB at pH 6.0. The thermodynamic parameters suggested that the adsorption process would occur naturally and exothermally. Pseudo-first-order, pseudo-second-order, and intraparticle diffusion models were used to test the adsorption kinetics of MO on **DCF**. The data were best fitted to the pseudo-second-order kinetic model. In conclusion, considering the first use of a calixarene-coated fabric as an adsorbent, the anionic–cationic dye selectivity of **DCF**, and the low cost and ease of application of the method, this study revealed important findings for further studies.

## Data Availability

Data created during this research can be accessed at: https://osf.io/89edc.

## Supporting Information Available

Estimation of point of zero charge (pH_PZC_) (Figure S1); variation of MO and RhB removal efficiency (%) at different solution pHs (Figure S2); Langmuir, Freundlich, and Temkin isotherms (Figure S3); effect of temperature on MO adsorption and Van’t Hoff plot for MO adsorption (Figure S4); effect of contact time, pseudo-first-order and pseudo-second-order kinetic models, and intraparticle diffusion kinetic model (Figure S5); breakup of preparation cost for novel adsorbent (Table S1) (PDF)

## Notes

This article does not contain any studies with animals or human participants performed by any of the authors.
