# Supplementary material for: Calixarene-Based Functional Fabric for Simultaneously Adsorptive Removal of Anionic and Cationic Dyes
Source: ACS Omega. 2024 Dec 24;10(1):181–92. doi: 10.1021/acsomega.4c04109 (PMC11740154; doi:10.1021/acsomega.4c04109)
Supplement: Supplementary file 1 — ao4c04109_si_001.pdf [file ao4c04109_si_001.pdf]

< Supplementary Material >

Calixarene-based functional fabric for simultaneously adsorptive removal of anionic and cationic dyes

Egemen Ozcelik[a], Begum Tabakci[b], Mustafa Karaman[a], Mustafa Tabakci[a,*]

[a]*Konya Technical University, Department of Chemical Engineering, 42250 Konya, Türkiye.*

[b]*Selçuk University, Department of Chemistry, 42130 Konya, Türkiye.*

Contents:

Fig. S1. Estimation of point of zero charge (pH_{PZC}) for **DCF**.

Fig. S2. The variation of MO and RhB removal efficiency (%) on **DCF** and unmodified fabric at different solution pHs.

Fig. S3. (A) Langmuir, (B) Freundlich, and (C) Temkin isotherms.

Fig. S4. (A) Effect of temperature on MO adsorption onto **DCF** and (B)Van't Hoff plot for MO adsorption onto **DCF**. Conditions: Initial concentration of solution, 2.5 mg/L. Solution pH, 6.0. Solution volume, 5.0 mL. Adsorbent dosage, 0.018 g. Contact time, 1.0 h.

Fig. S5. (A) Effect of contact time, inset: Pseudo-first-order and pseudo-second-order kinetic models, and (B) intra-particle diffusion kinetic model for adsorption of MO onto **DCF** (Co = 2.5 mg/L, t = 0-12 h, T = 25 ^{o}C, pH of 6.0, the mass of adsorbent = 0.018 g).

Table S1 Breakup of preparation cost for novel **DCF**.

*Corresponding author. Tel.: +90 332 2051924; fax:+90 332 2410635.
E-mail address: mtabakci@ktun.edu.tr (M. TABAKCI).

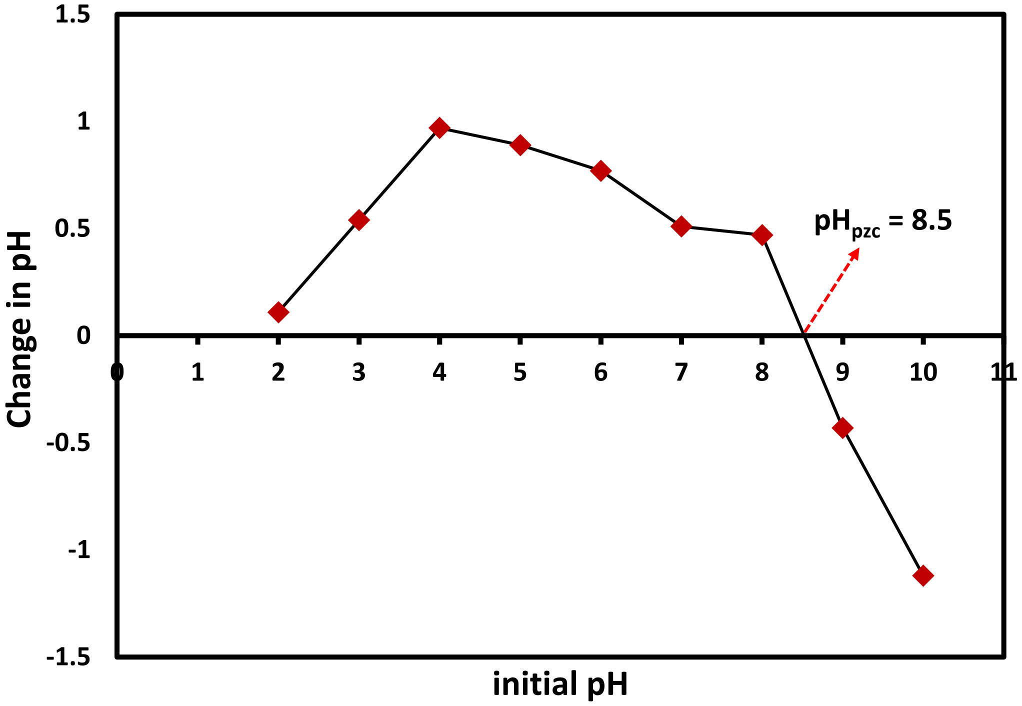

Fig. S1. Estimation of point of zero charge (pH_{PZC}) for **DCF**.

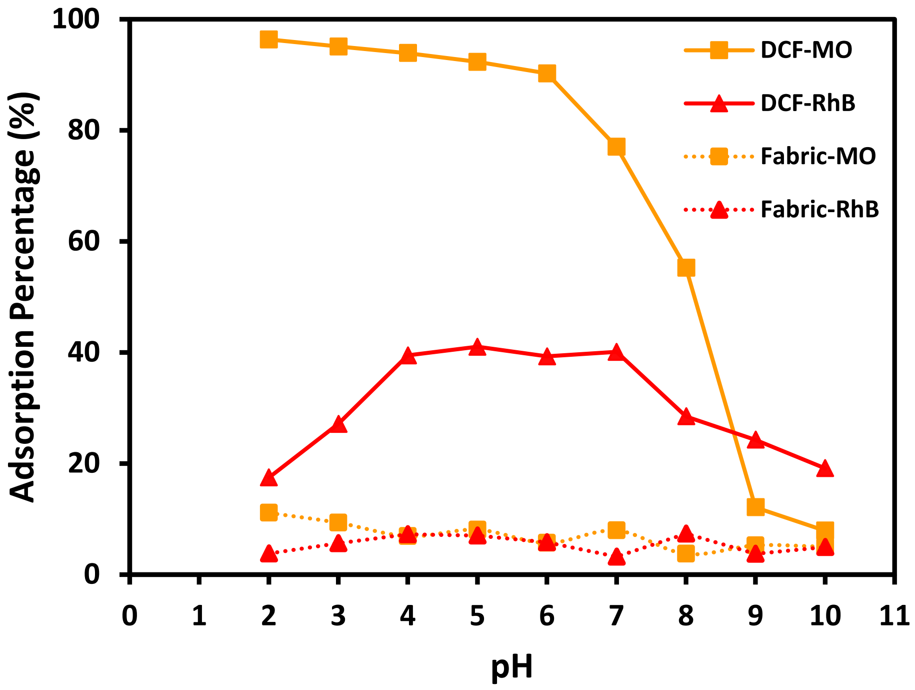

Fig. S2. The variation of MO and RhB removal efficiency (%) on **DCF** and unmodified fabric at different solution pHs.

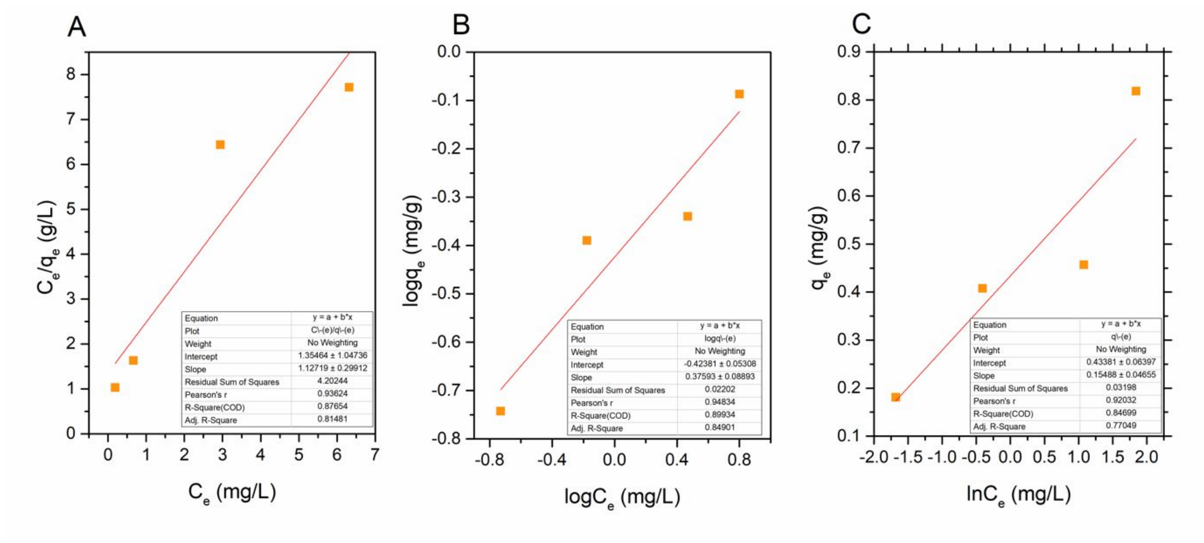

Fig. S3. (A) Langmuir, (B) Freundlich, and (C) Temkin isotherms.

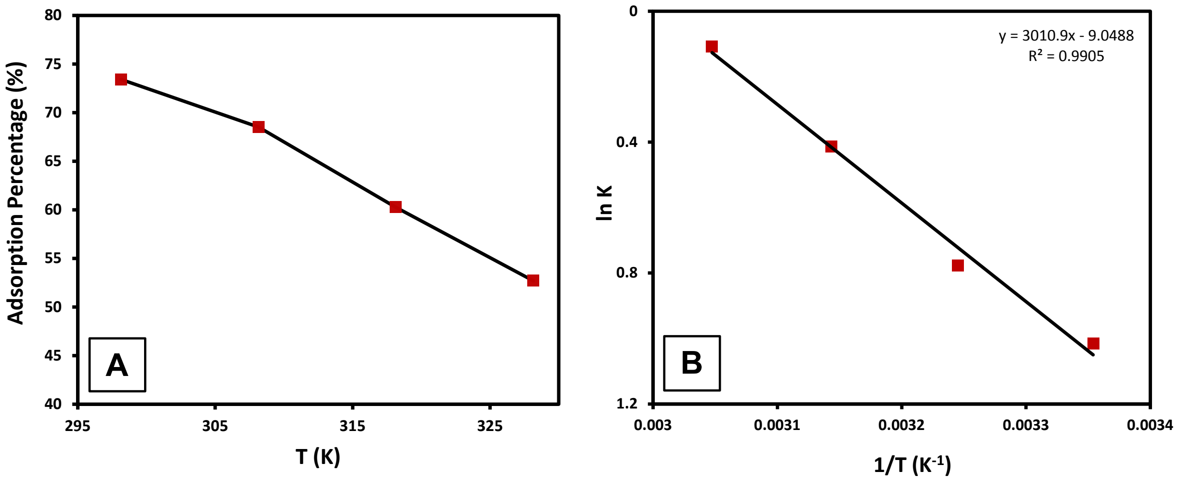

Fig. S4. (A) Effect of temperature on MO adsorption onto **DCF** and (B)Van't Hoff plot for MO adsorption onto **DCF**. Conditions: Initial concentration of solution, 2.5 mg/L. Solution pH, 6.0. Solution volume, 5.0 mL. Adsorbent dosage, 0.018 g. Contact time, 1.0 h.

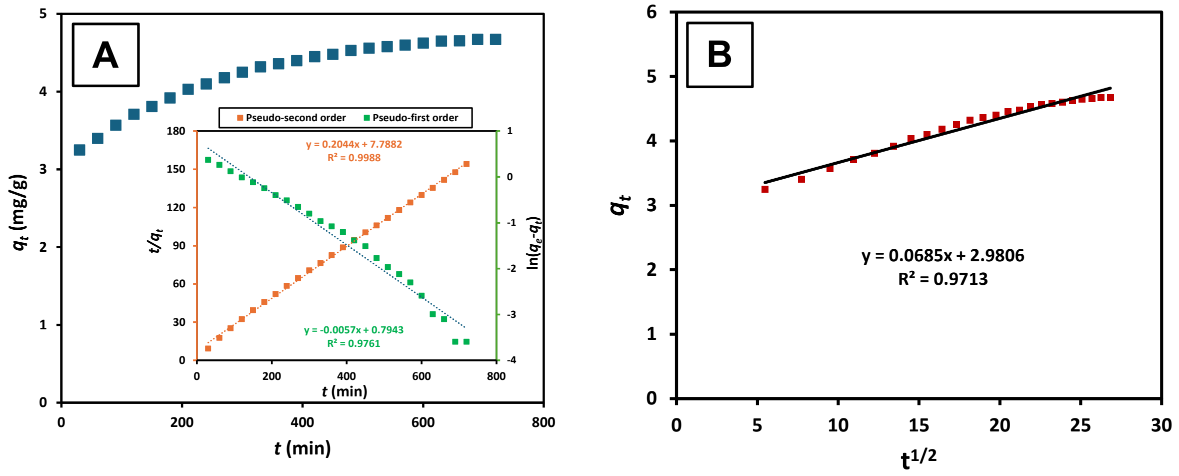

Fig. S5. (A) Effect of contact time, inset: Pseudo-first-order and pseudo-second-order kinetic models, and (B) intra-particle diffusion kinetic model for adsorption of MO onto **DCF** (C_o = 2.5 mg/L, t = 0-12 h, T = 25 ^{o}C, pH of 6.0, the mass of adsorbent = 0.018 g).

Table S1 Breakup of preparation cost for novel **DCF**.

**Particulars**	**Unit cost (TL)**	**Quantity used**	**Net cost (TL)**
p-tert-Butylphenol	50/500 g	9.16 g	0.93
Formaldehyde (37%)	37.5/500 mL	5.68 mL	0.43
Sodium Hydroxide	20/500 g	0.11 g	0.004
Diphenyl Ether	50/500 mL	73.26 mL	7.33
Ethyl Acetate	25/500 mL	137.4 mL	6.87
Acetic Acid	20/500 mL	18.32 mL	0.73
p-tert-Butylcalix[4]arene (1)		5.59 g	-
Aluminium chloride	25/500 g	3.79 g	0.19
Phenol	30/500 g	5.88 g	0.35
Toluene	50/500 mL	52.5 mL	5.25
Methanol	60/500 mL	39.1 mL	4.69
Calix[4]arene (2)		4.36 g	-
Acetic Acid	15/500 mL	12.40 mL	0.37
Dimethylamine (40% aqueous solution)	50/500 mL	6.97 mL	0.70
Formaldehyde	37.5/500 mL	4.09 mL	0.31
Tetrahydrofuran	40/500 mL	99.17 mL	7.93
Calix[4]arene (DMAM-Calix)		0.34 g	-
Sodium hydride (60% suspension in paraffin oil)	50/500 g	0.025 g	0.025
Toluene	15/500 mL	100 mL	3.00
Tetrabutylammonium Bromide	25/500 g	0.17 g	0.085
Acetone	20/500 mL	100 mL	4.00
Dimethylformamide	40/500 mL	100 mL	8.00
Glycidyl methacrylate	30/500 mL	10 mL	0.60
di-tert-Butyl peroxide (Luperox, 98%)	50/500 mL	10 mL	1.00
Silicon wafer (100 p-type)	50/piece	1x1 cm	5.00
Energy for all preparations	0.84/kwh	10 kwh	8.40
**Total cost**			**66.19**

Quantitiy of **DCF** obtained = ~1.0 g

Total cost for prepared **DCF**/g (as on 06.06.2024, 1.0 $ = TL 32.24) = **$ 2.05**
